# Property Enhancement of Waste Printed Circuit Boards Powders Reinforced Polypropylene by In Situ Magnesium Hydroxide Impregnation from Waste Lye

**DOI:** 10.3390/polym16060822

**Published:** 2024-03-15

**Authors:** Shenghui Tian, Jingwei Liu, Jiabao Gu, Chaoting Xie, Xiong Zhang, Xinlu Liu

**Affiliations:** 1 Chongqing Academy of Science and Technology, Chongqing 401331, China; 2 Provincial and Ministerial Co-Constructive of Collaborative Innovation Center for MSW Comprehensive Utilization, School of Metallurgy and Materials Engineering, Chongqing University of Science and Technology, Chongqing 401331, China; 2019004@cqust.edu.cn (J.L.); g17772378117@163.com (J.G.); 17823419565@163.com (C.X.); 18523038398@163.com (X.Z.); 17783561294@163.com (X.L.)

**Keywords:** waste printed circuit board powders, alkaline treatment, magnesium hydroxide, weathering resistance, flame retardant performance

## Abstract

Using alkali pretreatment can effectively remove residual variable-valence metals from non-metallic powder (WPCBP) in waste printed circuit boards. However, substantial amounts of waste lye are generated, which causes secondary pollution. On this basis, this study innovatively utilized waste alkali lye to prepare nano-magnesium hydroxide. When the dispersant polyethylene glycol 6000 was used at a dosage of 3 wt.% of the theoretical yield of magnesium hydroxide, the synthesized nano-magnesium hydroxide exhibited well-defined crystallinity, good thermal stability and uniform particle size distribution, with a median diameter of 197 nm. Furthermore, the in situ method was selected to prepare WPCBP/Mg(OH)_2_ hybrid filler (MW) and the combustion behavior, thermal and mechanical properties of PP blends filled with MW were evaluated. The combustion behavior of the PP/MW blends increased with the increasing hybrid ratio of Mg(OH)_2_, and the MW hybrid filler reinforced PP blends showed better thermal and mechanical properties compared to the PP/WPCBP blends. Furthermore, the dynamic mechanical properties of the PP/MW blends were also increased due to the improved interfacial adhesion between the MW fillers and PP matrix. This method demonstrated high economic and environmental value, providing a new direction for the high value-added utilization of WPCBP.

## 1. Introduction

With the continuous development of the electronics industry, the output of electronic waste (e-waste) increases progressively. It is estimated that 53.6 million tons of e-waste is generated globally per year and this figure is expected to double by 2050 [1,2]. Waste printed circuit boards (WPCBs) are an important byproduct in the disposal process of e-waste [3]. Nowadays, extensive studies have been carried out on the recovery of metals from electronic waste due to high-value-added issues [4,5]. However, a large amount of non-metallic components (broken glass fiber and thermosetting resin powder, referred to as WPCBP) from WPCBs cannot be effectively utilized, which not only causes resource waste but also results in significant environmental pollution [6,7]. According to recent domestic and international development trends, the most effective way to eliminate environmental pollution caused by WPCBP and achieve high-value-added utilization is selecting WPCBP as a filler in polymer composites [8,9]. However, the small amount of residual copper in WPCBP leads to a noticeable deterioration in the aging resistance of polyolefin composites, which seriously affect its secondary use [10,11]. The key scientific issue for the successful application of WPCBP in polymer composites is how to remove the residual copper from the WPCBP to improve the aging resistance of the composite material [12,13]. Traditional chemical methods for treating copper are mainly ammonia liquid dissolution, alkali liquid leaching, acid with an oxidant method, etc. [14,15]. However, these methods generate a large amount of waste liquid during the pretreatment process [15], causing serious secondary pollution, thereby restricting their industrial application. Therefore, it is urgent to investigate the pollution-free or low-pollution copper removal processes [16].

An NaOH/hydrogen peroxide micro-erosion system was successfully selected to remove the residual copper from WPCBP, which has the advantages of a constant copper micro-etching rate and a high copper-holding capacity. The oxidative induction period of the polypropylene (PP) composite material modified by the alkaline-treated WPCBP was significantly increased, leading to a notable improvement in the aging resistance of the PP blends. However, a massive amount of waste lye is generated during the copper removal process, which causes secondary severe pollution. Achieving efficient copper removal with minimal pollution has become an urgent issue to address. Building upon this foundation, this paper proposed using magnesium salt precipitants to titrate the waste lye to prepare of nano-magnesium hydroxide. This method addressed the secondary pollution caused by waste lye and produced nano-magnesium hydroxide, which served as an excellent flame retardant and smoke suppressant for polymeric blends [17]. It could be extensively applied in the production of cables, wires, transportation pipelines and household appliances, thereby eliminating secondary pollution from copper removal waste solutions and fully realizing the high-value reuse of resources.

This study systematically investigated the optimal synthesis conditions for producing nano-magnesium hydroxide from waste lye through particle size distribution analysis, thermogravimetric analysis, transmission electron microscopy analysis and scanning electron microscopy analysis. Then, three different hybrid ratios of WPCBP/Mg(OH)_2_ hybrid materials (MW) were prepared by in situ magnesium hydroxide impregnation from waste lye, and the physicochemical properties of the different MW hybrids were studied. Moreover, the combustion performance, thermal properties and mechanical properties of the different PP/MW blends were systematically investigated in this work.

## 2. Experimental Section

### 2.1. Raw Materials

The following raw materials were used:

Polypropylene Resin: PPH-T03, with an isotactic index ≥ 96.0 and Melt Flow Rate (MFR) of 3.0 (±0.5) g/10 min, produced by Sinopec Maoming Petrochemical Co., Ltd. (Guangzhou, China);

WPCBP: Powder form, 80 mesh, supplied by Jintian Enterprise Co., Ltd. (Guangzhou, China);

PP-g-MAH: Grafting rate of 1.0%, produced by Kingfa Science and Technology Co., Ltd. (Guangzhoug, China);

Reagents such as sodium hydroxide, magnesium chloride, anhydrous ethanol, hydrochloric acid, hydrogen peroxide and sodium dodecyl benzene sulfonate of analytical grade, purchased from Guangzhou Chemical Reagent Factory (Guangzhou, China).

### 2.2. Sample Preparation

#### 2.2.1. Modification of WPCBP

**Alkaline Treatment of WPCBP**. The WPCBP was placed in an oven at 105 °C for 2 h, then removed and sieved through an 80-mesh sieve for later use. A certain amount of solid potassium hydroxide was weighed to prepare a 10% potassium hydroxide dilute solution. Next, 50 g of the prepared WPCBP was added to a three-necked flask. A certain amount of the dilute potassium hydroxide solution and hydrogen peroxide was added. Finally, distilled water was added to maintain a solid–liquid ratio of about 1:5. The mixture was reacted under stirring at 300 r/min for 3 h. After filtration, the filtrate was retained. The resulting precipitate was then treated with a dilute hydrochloric acid solution to adjust the pH to around 4 and was reacted under stirring at 300 r/min for 0.5 h. After filtration and drying, the alkali-treated WPCBP was obtained. 

**Preparation of nano-Mg(OH)_2_ from waste lye**. The process for preparing model products of nano-Mg(OH)_2_ from waste lye was as follows: A certain amount of waste lye generated during the alkaline treatment of the WPCBP was taken and placed in a three-necked flask. Different amounts of the surfactant PEG-6000 were added and stirred evenly (the stirring speed was set to 200 r/min throughout the reaction). The reaction temperature was controlled, and a 1 mol/L MgCl_2_ solution was slowly added until the system reached a certain pH value. The reaction was continued for 1 h after the completion of the dropwise addition. The reaction mixture was allowed to age for 24 h, then centrifuged, washed and freeze-dried, and other processing steps were carried out to obtain Mg(OH)_2_. 

**Figure 1:**
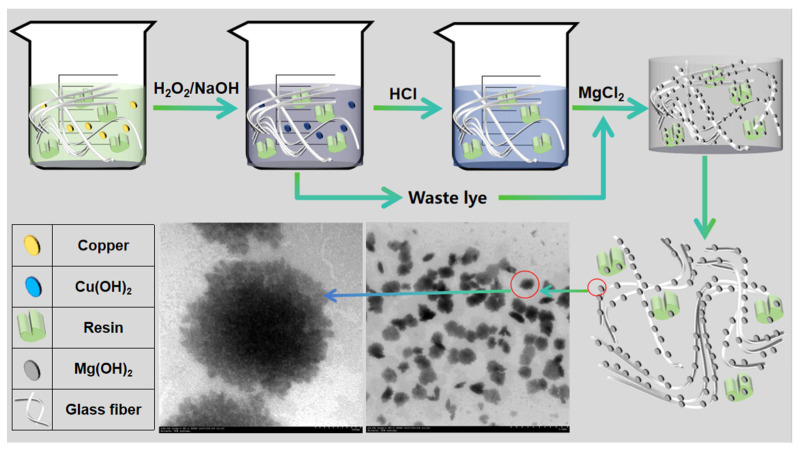
Schematic of WPCBP in situ magnesium hydroxide impregnation from waste lye.

**In situ magnesium hydroxide impregnation of WPCBP from waste lye**. The schematic diagram of the in situ generation is shown in Figure 1. The waste lye generated during the alkaline treatment process was placed in a 250 mL three-necked flask. Different amounts of the surfactant PEG-6000 and a certain amount of the WPCBP were added. The mixture was stirred evenly (the stirring speed was set to 200 r/min throughout the reaction). The reaction temperature was controlled, and a 1 mol/L MgCl_2_ solution was slowly added until the system reached the set pH value. After the addition of the MgCl_2_ solution was complete, the reaction continued for 1 h. After the reaction, the mixture was aged for 24 h. Finally, the WPCBP/Mg(OH)_2_ hybrid material (MW) was obtained after centrifugation, washing and freeze-drying. Three different magnesium hydroxide/WPCBP hybrids were prepared based on the calculated theoretical amount of Mg(OH)_2_, with ratios of 1:2, 1:1 and 2:1, respectively, and named MW0.5, MW1 and MW2, accordingly. 

#### 2.2.2. Preparation of PP Blends

PP, various WPCBP fillers and PP-g-MAH (20 wt.% of filler) were pre-mixed in specific proportions. They were then melted and blended on a two-roll mill (XKR-160A, Guangdong Zhanjiang Machinery Factory, Guangdong, Zhanjiang, China) at a controlled blending temperature of 160 + 3 °C for approximately 7 min. Subsequently, the mixture was subjected to a flat vulcanizing press (XLB-D, Zhejiang Hongtu Machinery Manufacturing Factory, Zhejiang, Ningbo, China) with a hot pressing temperature of 180 °C for 3 min, followed by a 7 min cooling press. The resulting sheets were then used to create standard test strips on a universal sample machine for testing.

### 2.3. Testing and Characterization

#### 2.3.1. Particle Size Analysis

Particle size analysis of the nano-Mg(OH)_2_ that was prepared from the waste lye was conducted on a BT-9300S laser particle size analyzer (Dandong Baite Instruments Co., Ltd, Liaoning, Dandong, China)with a Mie optical mode, and the medium’s refractive index was 1.333.

#### 2.3.2. Mechanical Property Testing

The mechanical property testing of the PP blends modified with in situ generated magnesium hydroxide from the WPCBP before and after thermal oxygen aging were conducted as follows:

Tensile tests were performed on a Z010 electronic universal testing machine from Zwick/Roell, Zwick, Ulm, Germany, according to the ASTM D638 standard [18], with a 20 mm/min testing speed;

Flexural tests were conducted on a Z010 electronic universal testing machine from Zwick/Roell, Zwick, Ulm, Germany, according to the ASTM D790 [19], with a span of 64 mm and a head speed of 20 mm/min;

Notched Izod impact tests were carried out on a 5113 digital impact tester from Zwick, Ulm, Germany, following the ASTM D256 [20] standard. 

In all cases, five specimens were tested and the average values were reported.

#### 2.3.3. Energy Dispersive X-ray Spectroscopy (EDS)

The waste lye prepared WPCBP/nano-magnesium hydroxide hybrid material was subjected to elemental analysis using the Quanta 200 environmental scanning electron microscope produced by FEI, Eindhoven, The Netherlands. The powder is evenly sprayed onto a single layer of flat mica sheet, and the mica sheet is adhered to the sample stage using conductive adhesive for testing. The testing voltage ranges from 10 KV to 15 KV, with one-time gold spraying.

#### 2.3.4. Dynamic Mechanical Analysis (DMA)

The dynamic thermomechanical properties of the WPCBP in situ generated magnesium hydroxide hybrid modified PP composite material were tested on the Netzsch 242C DMA. The testing mode was in the bending mode, with sample dimensions of 55 mm × 10 mm × 4 mm. The testing frequency was 1 Hz. The temperature was increased from 20 °C to 120 °C at a heating rate of 3 °C/min. The testing atmosphere was nitrogen gas.

#### 2.3.5. Scanning Electron Microscope (SEM) Analysis

The microscopic morphology analysis of the Mg(OH)_2_, MW and different PP blends were carried out with a Quanta 200 environmental scanning electron microscopy machine (FEI, The Netherlands) with a voltage of 10 kV~15 kV.

#### 2.3.6. X-ray Diffraction Analysis (XRD)

The nano-Mg(OH)_2_ was subjected to X-ray diffraction analysis using the X’Pert PRO X-ray diffractometer manufactured by the Dutch company PANalytical, Almelo, Netherlands, heart 1. The testing parameters were as follows: Cu target, scanning step size of 0.033, dwell time of 10 s and scanning range of 5° ≤ 2θ ≤ 90°.

#### 2.3.7. Limiting Oxygen Index Test (LOI)

The limiting oxygen index refers to the minimum volume fraction of oxygen, expressed as a percentage, at which a tested sample can sustain combustion in a mixture of nitrogen and oxygen under specified experimental conditions. The LOI of the WPCBP in situ generated magnesium hydroxide modified polypropylene composite material was tested with sample dimensions of 80 mm × 10 mm × 4 mm.

#### 2.3.8. Horizontal Combustion Test

The horizontal combustion performance of different PP blends was conducted using the horizontal and vertical burning apparatus produced by FTF in the UK, following the standard ASTM D635 [21]. During the experiment, the sample is fixed with dimensions of 130 mm × 13 mm × 4 mm and is vertically clamped in the fixture. Two 10 s flames are applied consecutively at the lower right end of the sample. The self-extinguishing time of the sample is recorded with a stopwatch, and the combustion quantity of the sample is measured. The horizontal burning rate (V_HB_) is then calculated to compare the combustion performance of the WPCBP in situ generated magnesium hydroxide-modified polypropylene composite material.

#### 2.3.9. Transmission Electron Microscope Analysis (TEM)

The morphology of the nano-magnesium hydroxide prepared from waste lye was observed using a Japanese Hitachi HF-3300 transmission electron microscope, Tokyo, Japan, with an acceleration voltage of 80 KV. The testing sample was prepared as follows. The ultrasonically dispersed nano-magnesium hydroxide powder suspension was diluted to a concentration between approximately one-thousandth to one-five-thousandth using distilled water. The suspension was drawn onto a 200-mesh copper grid using a pipette and a 3 wt.%. Phosphotungstic acid solution was used as a staining agent for negative system staining. After air-drying by natural sedimentation, the morphology of the nanoparticles was observed using the transmission electron microscope.

## 3. Results and Discussion

### 3.1. Physicochemical Properties of Mg(OH)_2_

An NaOH/hydrogen peroxide micro-erosion system was selected to pretreat the WPCBP to remove the residual copper. Table 1 presents the XRF data of ash content in the WPCBP before and after the alkali treatment. From the table, it can be observed that the content of the variable-valent metal copper in the WPCBP that was treated with a 10 wt.% sodium hydroxide solution significantly decreased. This indicated that the alkali treatment of the WPCBP had achieved a good copper removal effect. However, much waste lye was generated during the copper removal process, leading to serious secondary pollution. Based on this, this study proposed using magnesium salt precipitant to titrate the waste lye to prepare nano-magnesium hydroxide. This method not only effectively addressed the issue of secondary pollution caused by the waste lye but also produced nano-magnesium hydroxide, a commonly used polyolefin flame retardant, which has high economic and environmental value [9,12].

During the synthesis of the magnesium hydroxide, the reaction pH value and the dosage of dispersant PEG significantly influenced its particle size, structure and morphology. Some scholars researched the reaction pH value for synthesizing magnesium hydroxide. The results indicated that the isoelectric point of Mg(OH)_2_ particles was around pH 9.5. When the pH value of the synthesis reaction was higher than the isoelectric point pH, the surface of the Mg(OH)_2_ particles became negatively charged. The surface potential also rose with increasing pH value, leading to more pronounced repulsion between the particles. This resulted in the preparation of uniform-sized Mg(OH)_2_ powder. In this study, the waste lye generated during the alkali treatment process was first enriched and then treated with a magnesium chloride solution. A series of magnesium hydroxide powders were prepared by controlling the dosage of PEG-6000 under a fixed reaction pH value of 11. The physicochemical properties of the magnesium hydroxide powders prepared with different amounts of PEG-6000 were characterized using particle size analysis, scanning electron microscopy, transmission electron microscopy, X-ray diffraction analysis and thermogravimetric analysis. The optimal dosage of PEG-6000 in the synthesis of nano-magnesium hydroxide was discussed.

#### 3.1.1. Particle Size Analysis

**Figure 2:**
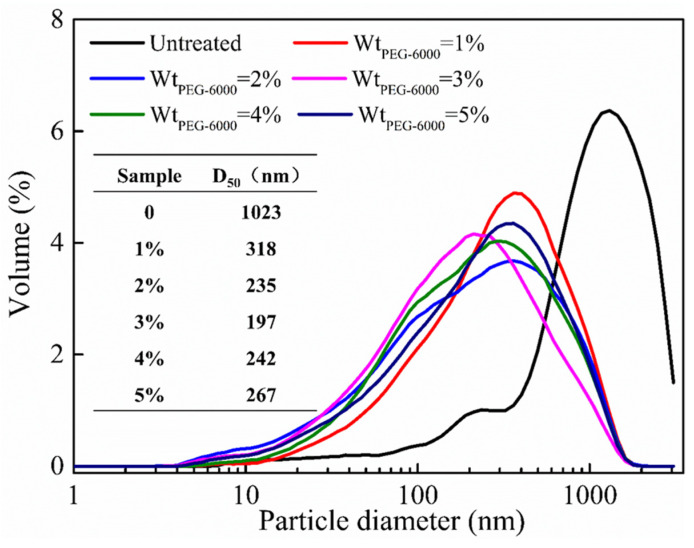
The impact of different PEG dosages on the particle size distribution of magnesium hydroxide and corresponding median diameter (D_50_) data.

In this study, particle size analysis was conducted on the magnesium hydroxide powders prepared with different amounts of PEG-6000. Figure 2 shows the influence of the different PEG dosages on the particle size distribution of the magnesium hydroxide and the corresponding median diameter (D_50_) data. The median diameter (D_50_) was defined as the measured powders having 50 wt.% of the particle smaller than this particle size, which was selected as an essential parameter to evaluate the average particle size of the powders.

From Figure 2, it can be observed that the particle size distribution of the magnesium hydroxide powder without PEG-6000 modification was the widest, with a median diameter larger than 1 μm. The measured particle size of the powder could not reach the nanoscale level. However, the particle size of the magnesium hydroxide powder treated with PEG-6000 significantly decreased, with median diameters all below 500 nm. This was likely to be because magnesium hydroxide had many hydroxyl groups on its surface, making it hydrophilic and prone to aggregation. PEG-6000, which also contains a large number of hydroxyl groups in its structure, could interact with the hydroxyl groups on the surface of magnesium hydroxide during the reaction process, reducing the probability of particle collision and aggregation. Therefore, the addition of PEG-6000 could significantly reduce the particle size of magnesium hydroxide.

From the corresponding median diameter results in Figure 2, it can be observed that when the dosage of PEG-6000 was 3 wt.% of the theoretical yield of magnesium hydroxide, the obtained particle size of the magnesium hydroxide was the smallest, with a median diameter of 197 nm. However, when the dosage of PEG-6000 was too high, the median diameter of the powder increased. This might have been due to the excessive addition of PEG-6000, whose distinctive long-chain structure had impacted the dispersion effect of the powder. Therefore, the optimal dosage of PEG-6000 was determined to be 3 wt.% of the theoretical yield of magnesium hydroxide. In the following sections, the study continues with the characterization of the magnesium hydroxide powders prepared in the unmodified system, as well as in the system modified with 3 wt.% PEG-6000.

#### 3.1.2. XRD Analysis

**Figure 3:**
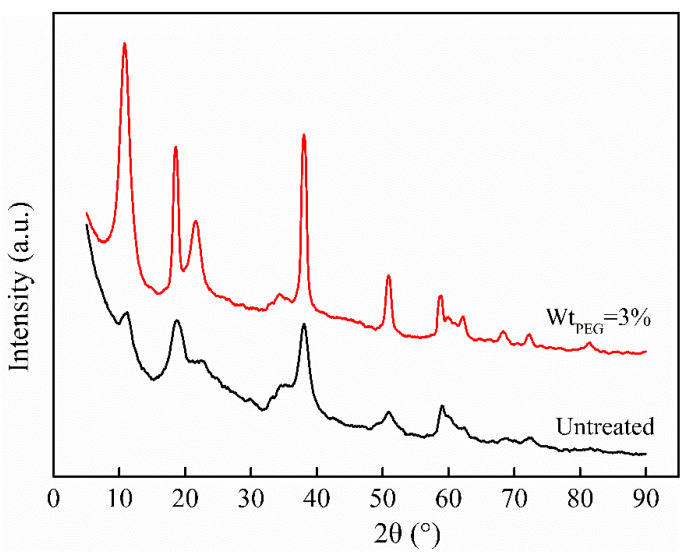
XRD patterns of different magnesium hydroxide powders.

Figure 3 presents the XRD patterns of two types of magnesium hydroxide powders. It can be observed from the figure that the diffraction peaks of both powders are closely matched with the standard PDF card of magnesium hydroxide, with a high repetition rate of 90%. This confirmed the synthesized product as magnesium hydroxide. Compared to the unmodified system, the diffraction peaks of the powder in the PEG-6000-modified system were more pronounced. This indicated that the magnesium hydroxide synthesized in this system had a more complete crystallization [22]. This was consistent with previously reported results. Under low-temperature and atmospheric pressure conditions, the PEG-6000 not only played a role in controlling the particle size of the synthesized nano-magnesium hydroxide powder but also, by affecting the size, shape, structure and growth rate of the grains during the synthesis process, led to the preparation of magnesium hydroxide powder with a more complete crystallization [23].

#### 3.1.3. TGA Analysis

**Figure 4:**
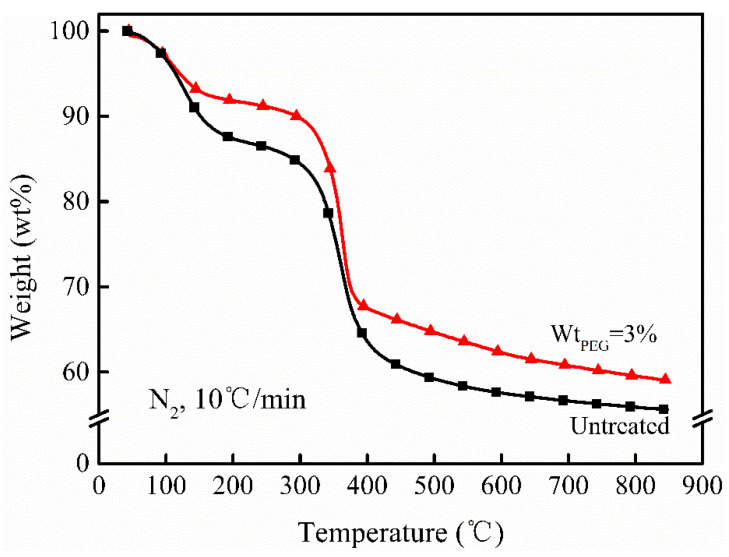
Thermogravimetric analysis curves of different magnesium hydroxide powders.

Since magnesium hydroxide can be used as a flame retardant and smoke suppressant in plastics, it was necessary to analyze its thermal stability. This study continued to conduct the thermogravimetric analysis of the two types of magnesium hydroxide powders. The results are shown in Figure 4 and Table 2. T_onset_ was defined as the temperature of the powder at a weight loss of 10 wt.%, while the char residue represented the char yield of the powder at 900 °C. The thermogravimetric analysis results indicated that the thermal stability of the magnesium hydroxide synthesized in the PEG-6000 system was superior to the unmodified system. The powder had a higher initial decomposition temperature and char yield. This was attributed to the crystallinity of the magnesium hydroxide synthesized in the PEG-6000 system being better than the untreated system. The powder contained less crystalline water, resulting in improved thermal performance.

Through calculations, it was determined that the theoretical char yield of the magnesium hydroxide powder was approximately 70 wt.%, significantly higher than the char yields of the two types of magnesium hydroxide powders synthesized in this study (as shown in Table 2). This was likely to be because the magnesium hydroxide powder synthesized with PEG-6000 contained some crystalline water and PEG-6000, resulting in a lower char yield. On the other hand, the magnesium hydroxide powder in the unmodified system had imperfect crystallization, leading to a higher content of crystalline water and thus an even lower char yield.

#### 3.1.4. SEM Analysis

**Figure 5:**
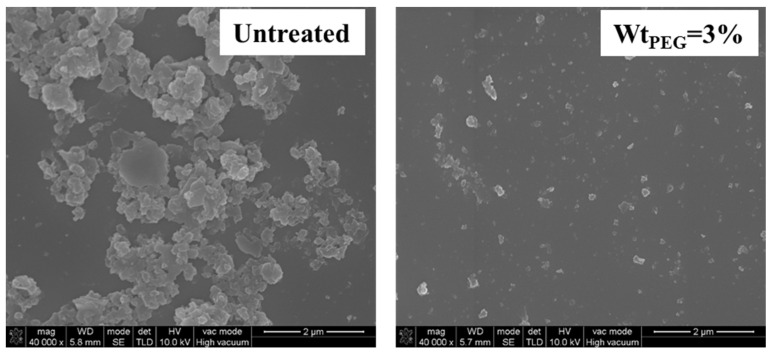
The influence of surfactant on the microscopic morphology of magnesium hydroxide (magnification: 40,000).

Figure 5 displays the SEM images of the two types of magnesium hydroxide powders, both magnified at 40,000 times. From the images, it can be observed that the unmodified magnesium hydroxide powder exhibited severe aggregation, with particles densely stacked in layers, indicating poor dispersion performance and, consequently, a larger particle size. However, in the SEM image of the magnesium hydroxide powder synthesized with PEG-6000, the particles were evenly distributed without significant signs of aggregation. Additionally, the powder had a smaller particle size, consistent with the results from the particle size analysis in Section 3.1.1. Adding the PEG-6000 effectively reduced the particle size of the nano-magnesium hydroxide powder.

#### 3.1.5. TEM Analysis

**Figure 6:**
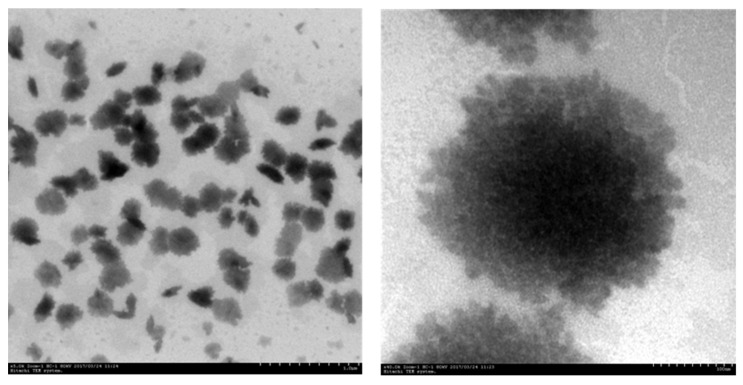
TEM image of 3 wt.% PEG-6000 generated magnesium hydroxide.

Figure 6 shows the transmission electron microscope (TEM) image of the nano-magnesium hydroxide powder synthesized with PEG-6000. From the image, it can be observed that the nano-magnesium hydroxide particles prepared with 3 wt.% PEG-6000 were uniformly distributed, with a consistent size and plate-like morphology. In the TEM magnification image, it is also possible to observe a small number of extremely small particles adsorbed onto the surface of the powder, indicating that the crystallinity of the synthesized magnesium hydroxide powder was not perfect, leading to poorer thermal stability. Magnesium hydroxide has three crystal forms: needle-like, rod-like and plate-like. The crystal form is related to parameters such as dispersant and pH value. PEG-6000 might have promoted the plate-like crystallization of the magnesium hydroxide. Furthermore, from Figure 6, it can be seen that the particle size of this nano-magnesium hydroxide was approximately 200 nm, which is consistent with the results of the particle size analysis in Section 3.1.1. This indicates that the addition of 3 wt.% PEG-6000 could lead to the preparation of uniformly distributed plate-like magnesium hydroxide powder.

### 3.2. Physicochemical Properties of Different MW Hybrids

Two problems must be solved to achieve good results when WPCBP is used as a filler for polyolefin. Firstly, it was necessary to remove the residual copper. Then, proper surface modification of the WPCBP must be carried out to make it uniformly dispersed in polymer blends and form firm interface bonding. In previous research, the enrichment of the waste liquid generated during alkali treatment and treatment with magnesium chloride solution produced nano-magnesium hydroxide, which can be used as a flame retardant and smoke suppressant in plastics. This method eliminated secondary pollution from the treatment solution and further expanded resource utilization.

Hybrid fillers refer to hybrids formed by two particles of different structures or sizes through electrostatic adsorption, chemical bonding, hydrogen bonding, van der Waals forces, etc. Unlike single inorganic fillers, novel hybrid fillers typically exhibit superior synergistic effects in polymeric blends. Currently, these advanced hybrid fillers can be prepared through methods such as in situ growth, electrostatic self-assembly and chemical bonding. In the next sections of this research, this study continues to utilize the in situ growth method to recycle the waste lye generated during the alkali treatment process to generate magnesium hydroxide on the surface of WPCBP. By controlling the theoretical yield of magnesium hydroxide, three different magnesium hydroxide/WPCBP hybrids are prepared (designated as MW0.5, MW1 and MW2). Scanning electron microscopy was selected to characterize the microscopic morphology and elemental composition of these three hybrids.

#### 3.2.1. SEM Analysis of Different MW Hybrids 

**Figure 7:**
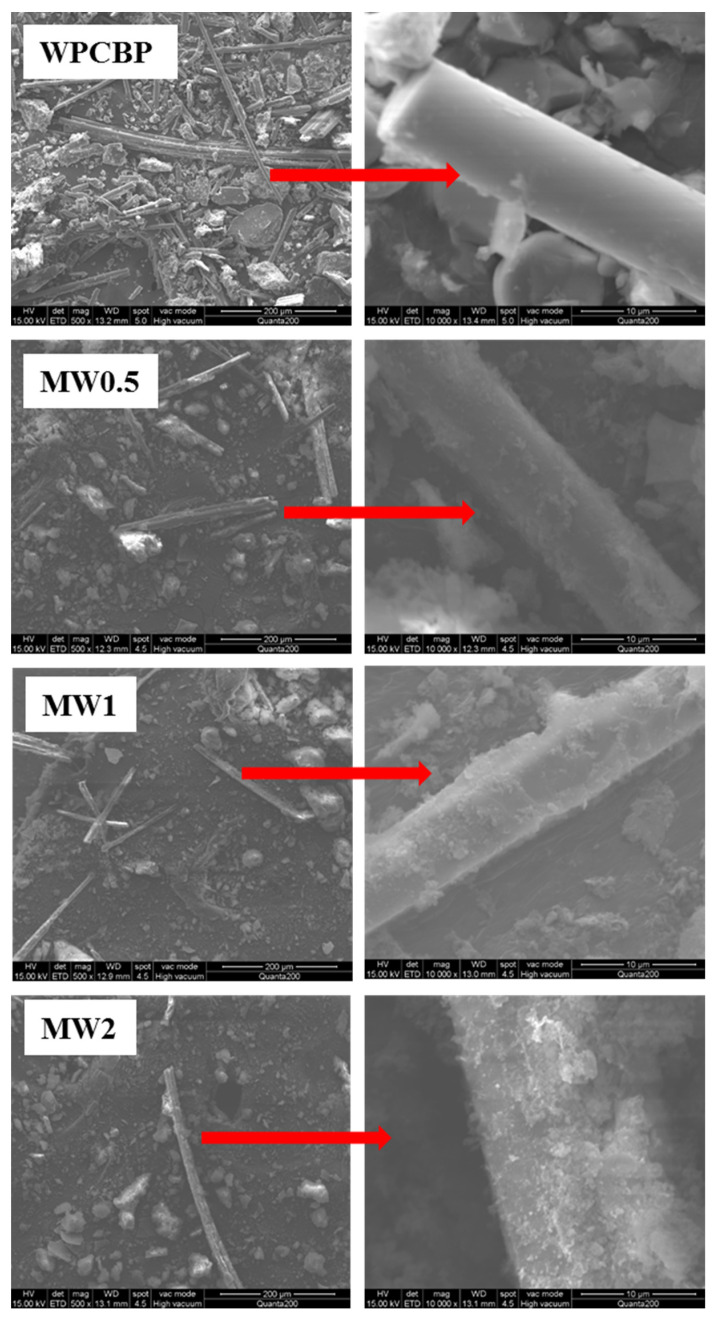
Microscopic morphology of different MW hybrids (magnification: 500).

The microscopic morphology of the magnesium hydroxide/WPCBP hybrids with different hybrid ratios is shown in Figure 7. From the images, it was evident that the surface of the unmodified WPCBP was covered with a certain amount of resin, and the surface of the glass fiber was smooth relatively without any noticeable protrusions. In contrast, the outlines of the resin and glass fiber in the three MW hybrids treated with waste lye became blurred. Granular substances can be observed adhering to the surface, and the surface of the powder became uneven and rough, indicating the formation of magnesium hydroxide powder.

It also can be observed that the amount of powder on the surface of the MW mixed filler significantly increased with the increase in the hybrid ratio of magnesium hydroxide/WPCBP. Among them, the texture of the MW2 was the roughest and magnesium hydroxide particles with a larger volume were stacked block-like on the surface of the WPCBP. From the corresponding SEM high-magnification image, it can be seen that the glass fibers in the MW2 were almost completely encapsulated by the magnesium hydroxide powder. In summary, with the change in the ratio of magnesium hydroxide to WPCBP, the surface morphology of the magnesium hydroxide/WPCBP hybrid underwent significant alterations. The magnesium hydroxide powder on the surface of the WPCBP noticeably increased as the hybrid ratio of magnesium hydroxide increased.

#### 3.2.2. EDS Analysis of Different MW Hybrids

**Figure 8:**
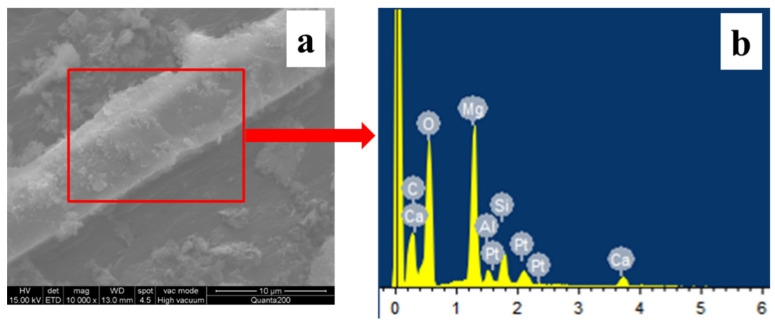
(**a**) Microscopic morphology of MW1 hybrid (magnification: 10,000); (**b**) EDS spectrum.

To further demonstrate the formation of magnesium hydroxide on the surface of the WPCBP, EDS spectroscopy was employed to analyze the composition of the MW1 hybrid, and the results are shown in Figure 8 and Table 3. The EDS analysis of the glass fiber and its surface powder, shown in Figure 8, indicated the absence of variable-valence metals such as copper, iron and nickel in the system, which suggested that variable-valence metals in WPCBP can be removed effectively through alkali treatment. Additionally, the EDS analysis data showed a significant increase in magnesium content in the powder, indicating that the hybrid powder on the surface of the glass fiber was definitely magnesium hydroxide.

### 3.3. Flame-Retardant Properties of Different PP Blends

Polypropylene is widely used in daily life because of its low price and good comprehensive performance as one of the universal plastics. Therefore, it was essential to study its flame-retardant properties. Nano-magnesium hydroxide powder prepared from waste lye can be used as a flame retardant and smoke suppressant for polypropylene. Therefore, in recent papers, limiting oxygen index and horizontal combustion rate have been used to study the flame-retardant performance of PP blends modified by different MW hybrids.

**Figure 9:**
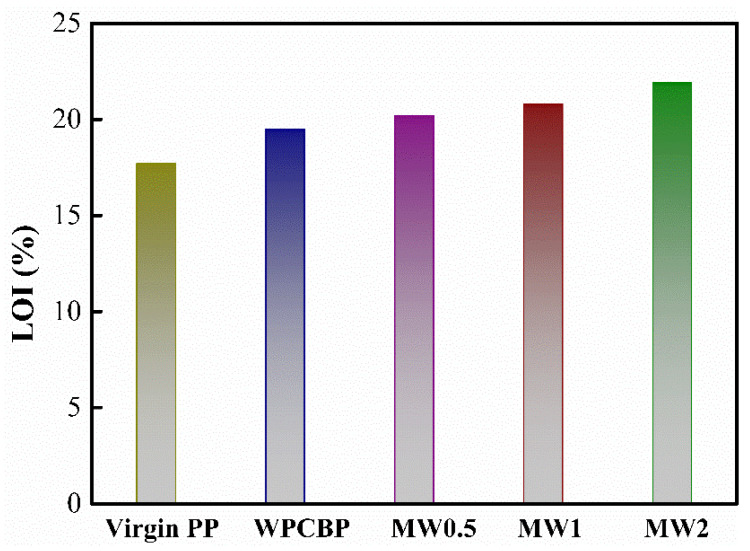
Influence of hybrids on the oxygen index of PP blends.

Figure 9 shows the influence of different hybrids on the oxygen index of the PP blends. It can be observed that the limiting oxygen index (LOI) of the PP blends was improved when filled with WPCBP compared to the pure PP blends. This may be because adding the WPCBP increased the inorganic components in the composite material. Additionally, the resin matrix of the WPCBP contained a small number of halogenated flame retardants, such as brominated epoxy resin, which contributed to a particular flame-retardant effect, increasing the oxygen index of the composite material. Furthermore, it can be seen from Figure 9 that the LOI of the PP blends filled with MW powder was further enhanced after hybrid modification. As the hybrid ratio of magnesium hydroxide increased, the LOI of the composite material also showed an upward trend. This was because magnesium hydroxide decomposed to produce water during the combustion process, which was an endothermic reaction. The thermal decomposition of magnesium hydroxide was conducive to forming a surface carbon layer, thus achieving a flame-retardant effect.

Table 4 shows the effect of the hybrids on the horizontal burning rate of the PP blends. It can be observed that the addition of the WPCBP significantly increased the burning rate of the PP blends. As mentioned in previous studies, WPCBP contains a small amount of halogenated flame retardants, which play a flame-retardant role when used in PP blends. However, due to the presence of a large amount of glass fiber bundles and individual glass fibers in WPCBP, PP blends filled with it are prone to exhibit a “candlewick effect” during combustion, accelerating the burning of the PP matrix and leading to an increase in the horizontal burning rate.

It can be observed from Table 4 that with the addition of the MW hybrids and an increase in the hybrid ratio, the horizontal burning rate of the PP blends decreased significantly, which enhances the combustion performance of the PP blends to some extent. This was because the magnesium hydroxide component in the hybrids, commonly used as a flame retardant for polyolefins, decomposes during combustion, producing water that absorbed a portion of the heat. Additionally, water vapor diluted the oxygen at the surface of the PP blends, thereby improving the combustion performance of the PP blends. Among them, the MW2 system had the highest content of nano-magnesium hydroxide, resulting in the lowest horizontal burning rate for the corresponding composite material, barely 18 mm/min.

### 3.4. Thermal Properties of Different PP Blends

**Figure 10:**
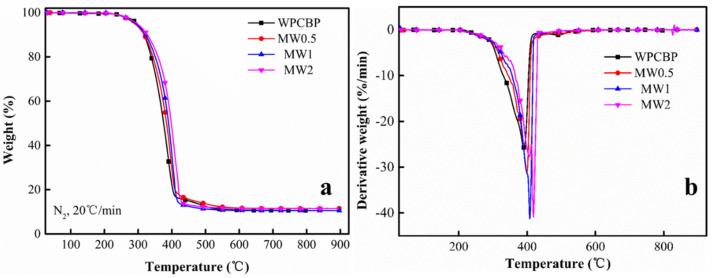
TG (**a**) and DTG (**b**) curves of PP blends modified with MW.

This study continued the analysis of the thermal stability of the PP blends modified with the MW hybrids prepared at different hybrid ratios. The results are shown in Figure 10 and Table 5. Here, T_onset_ was defined as the temperature at which the composite material loses 10 wt.% of its weight, T_max_ was defined as the temperature corresponding to the maximum rate of thermal weight loss, and char residue represented the residual carbon content of the composite material at 900 °C.

The initial decomposition temperature, maximum weight loss rate and residue at 900 °C for MW-modified PP blends under a nitrogen atmosphere had all increased compared to the unmodified WPCBP system. Among them, the MW2 system exhibited the best thermal stability. This result can be explained from two aspects. Firstly, the nano-magnesium hydroxide generated during the hybridization process increased the proportion of the inorganic phase in the system, leading to an improvement in the thermal stability of the filled and modified PP blends. The higher the hybridization ratio, the higher the amount of magnesium hydroxide generated in the corresponding hybrid filler, resulting in better heat resistance of the filled PP composite material. Secondly, the in situ generated magnesium hydroxide powder on the surface of the WPCBP effectively enhanced the interfacial bonding ability between the filler and the PP matrix. This strong interfacial bonding allowed for better transmission of thermal stress to the filler during heating, leading to a significant increase in both the initial decomposition temperature and the temperature of the maximum weight loss rate of the PP blends.

### 3.5. Mechanical Properties of Different PP Blends

**Figure 11:**
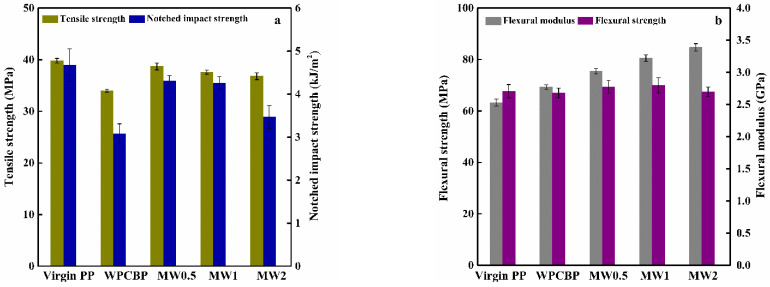
The impact of hybrid material on the mechanical properties of PP blends. ((**a**): Tensile strength and Notched impact strength, (**b**): Flexural strength and modulus).

Figure 11 illustrates the impact of different hybrid ratios of MW-modified PP blends on their mechanical properties. As seen in Figure 11a, in comparison to pure PP, the WPCBP affected the impact strength and tensile strength of the blends significantly, leading to a reduction in strength. This was attributed to the layer of thermosetting resin encapsulating the glass fibers in the WPCBP. The compatibility of this interface with the PP matrix could be better, causing internal stresses generated during loading to not transmit efficiently to the WPCBP. Instead, they propagated along the surface of the glass fibers, resulting in cracks. As a consequence, the impact resistance of the PP blends experienced a significant decrease.

Moreover, as observed in Figure 11, various mechanical properties of the PP/MW composite system showed varying degrees of improvement with the progress of the hybridization reaction. Additionally, with an increase in the hybridization ratio, the tensile strength, impact strength and flexural modulus of the PP/MW blends exhibited a trend of initially increasing and then decreasing. At the same time, the flexural modulus continued to rise. This was attributed to the in situ generation of magnesium hydroxide loaded onto the surface of the WPCBP uniformly during the hybridization process. This increased the specific surface area of the WPCBP and effectively formed a serrated structure at the contact surface between the WPCBP and PP. When subjected to external forces, this irregular surface can impede crack propagation effectively, reducing the occurrence of large cracks along the surface of the glass fibers during external force application. As a result, the mechanical properties of the PP blends experienced a significant enhancement. However, when the hybridization ratio of magnesium hydroxide to WPCBP in the hybrid material was excessively high, the generated magnesium hydroxide had a larger particle size. It tended to agglomerate on the surface of the WPCBP. Consequently, the hybrid interface between the hybrid material and PP blends could not form a serrated interface effectively. Moreover, due to the larger particle size of magnesium hydroxide, it was prone to form defects, leading to stress concentration. This resulted in a decrease in the mechanical properties of the composite material.

### 3.6. Dynamic Mechanical Properties of Different PP Blends

**Figure 12:**
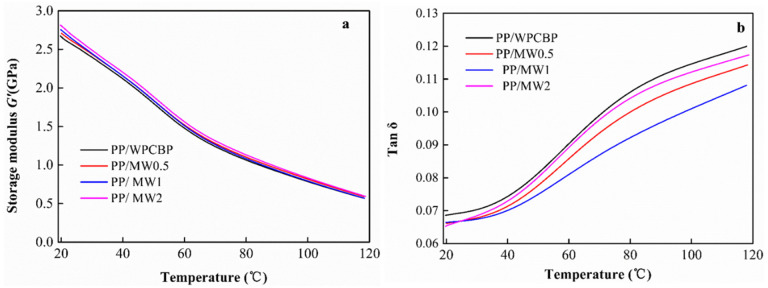
Influence of hybrid material on dynamic mechanical properties of PP blends: (**a**) Storage modulus E’, (**b**) Loss factor tan δ.

The dynamic mechanical properties of the PP blends modified by the MW hybrids were further investigated and the results are shown in Figure 12. Figure 12a illustrates the influence of the hybrid bodies on the storage modulus (G’) of the PP blends. It can be observed from the figure that the storage modulus (G’) of all four PP blends decreased with increasing temperature across the entire temperature range. Among them, the MW system modified by hybridization showed higher storage moduli than the unmodified system. This indicated that the magnesium hydroxide in the MW hybrid body formed a serrated interface with the PP matrix, effectively improving the interface bonding performance between the WPCBP and the PP matrix at lower temperatures, thereby increasing the stiffness of the composite material and enhancing its storage modulus (G’).

Figure 12b shows the influence of the hybrid material on the loss factor Tanδ of the PP composite material. It can be clearly seen that the Tanδ of the four PP blends all exhibited an increasing trend within the entire experimental temperature range. At the same temperature, the unmodified WPCBP system had the highest Tanδ value, followed by the MW2 system, and the PP composite material system filled with MW1 had the lowest Tanδ value. This was consistent with the results of the study on the static mechanical properties of the hybrid modified PP blends in Section 3.5, indicating that in situ generated magnesium hydroxide on the surface of WPCBP can form a sawtooth-like structure with the PP matrix under a certain hybrid ratio, thereby enhancing the dynamic mechanical properties of PP blends.

### 3.7. Morphology Analysis of Different PP Blends

**Figure 13:**
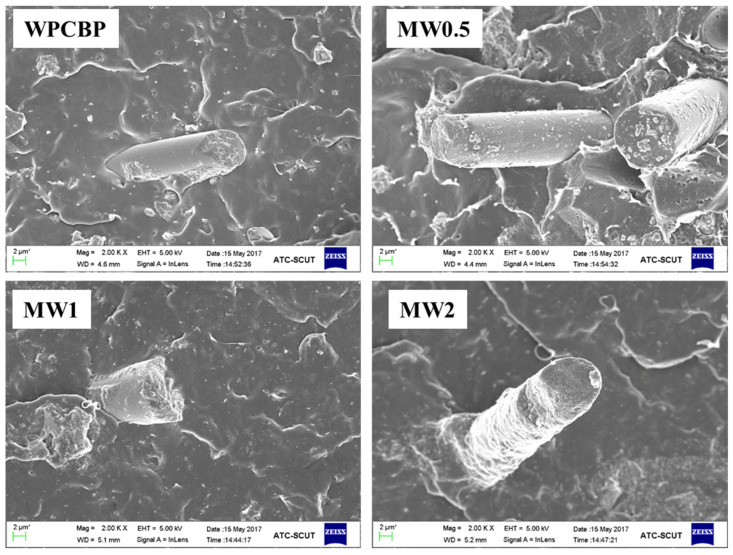
Microscopic morphology of hybrid modified PP blends (magnification: 2000×).

From the mechanical properties of the blends studied so far, the presence of magnesium hydroxide resulted in significant changes in the properties of the PP blends, which could be attributed to the improved interaction of the filler and PP matrix. SEM was selected to examine the microstructure of the PP blends, and the results are shown in Figure 13. It could be observed that the surface of the untreated WPCBP was very smooth, which had a flat contact surface with the PP matrix. Additionally, there was evidence of glass fiber pull-out in the powder. This indicated a relatively poor interface bonding between the WPCBP filler and the PP matrix. When the composite material was subjected to external forces, the stress could not be effectively transmitted, which resulted in the formation of cracks on the surface of the WPCBP and showed a decrease in the mechanical properties of the PP blends.

Furthermore, it could also be observed that the impact fracture surface of the WPCBP in situ generated magnesium hydroxide system was rough, with granular material at the interface between the filler and the matrix, forming a serrated contact surface. Most of the hybrid fillers were embedded in the PP matrix. This result was consistent with the mechanical properties of the hybrid modified PP blends in Section 3.5 and Section 3.6. After hybrid modification, the interface bonding performance between the MW and the matrix was significantly improved [14]. When the composite material was subjected to external forces, the stress was transmitted to the WPCBP through the magnesium hydroxide, leading to a significant improvement in performance of the PP blends. With the increase in the hybrid ratio, it can be observed that the magnesium hydroxide powder was not well dispersed in the PP, resulting in agglomeration, which led to a decrease in the impact performance of the PP blends. 

## 4. Conclusions

Alkaline treatment of the WPCBP effectively removed the multivalent transition metals from the WPCBP, which accelerated the oxidative degradation of the PP/WPCBP blends. However, the alkaline process generated a large amount of waste lye, which resulted in secondary pollution. This study innovatively utilized the waste lye generated during the alkaline treatment of the WPCBP to prepare nano-magnesium hydroxide, and the physicochemical properties of the synthesized nano-magnesium hydroxide were characterized through particle size analysis, XRD and TEM. The results showed that the optimal amount of dispersant PEG-6000 was 3 wt.% of the theoretical yield of magnesium hydroxide, and the synthesized nano-magnesium hydroxide exhibited well-defined crystallinity, good thermal stability, uniform particle size distribution and a median diameter of 197 nm. This method not only effectively addresses the issue of secondary pollution caused by waste lye but also produces nano-magnesium hydroxide, a high-value-added flame retardant that is widely used in plastics. 

Furthermore, this study successfully loaded magnesium hydroxide onto the WPCBP using an in situ generation method, prepared a novel MW hybrid filler and systematically investigated the influence of different hybridization ratios of MW on the combustion and thermal properties of the PP blends with a fixed filler content of 20 wt.%. The LOI test and horizontal burning test showed a significant increase in LOI values and a noticeable decrease in horizontal burning rate for PP blends filled with MW. Moreover, as the hybridization ratio of the MW increased, the flame-retardant performance of the blends also improved. The TGA results indicated that the onset decomposition temperature, temperature of maximum weight loss and char residue of the MW-filled PP blends all increased, which indicated a remarkable enhancement in the thermal stability of the PP blends. The results of the mechanical property tests indicated that the various mechanical properties of the MW-filled PP blends after hybrid modification were significantly improved compared to the unmodified WPCBP system, and the SEM results also demonstrated that the magnesium hydroxide particles hybridized on the surface of the WPCBP formed a serrated interface between the WPCBP and the PP matrix. Better mechanical and flame-retardant performance obtained through a state approach will help to broaden the application of WPCBP in PP blends, and provide a promising way to recycle waste for a sustainable world.

## Figures and Tables

**Table 1:** XRF Data of ash content in WPCBP before and after alkali treatment.

Sample	Content (wt.%)							
	SiO_2_	CaO	Al_2_O_3_	MgO	CuO	Fe_2_O_3_	Cr_2_O_3_	Others
WPCBP	52.7	20.7	14.8	1.0	2.1	1.1	0.9	6.7
Alkali-treated WPCBP	60.1	19.3	12.9	0.6	0.4	-	0.2	6.5

**Table 2:** Thermogravimetric analysis data of different magnesium hydroxide powders.

Sample	T_onset_ (°C)	Char Residue (wt.%)
Untreated Mg(OH)_2_	151.4	55.6
PEG treated Mg(OH)_2_	294.7	59.1

**Table 3:** Related EDS data of MW1 hybrid.

Element	C	O	Mg	Al	Si	Ca	Pt
Per. (wt.%)	19.39	49.05	17.93	1.63	3.95	2.23	5.82

**Table 4:** Impact of hybrids on the horizontal burning rate of PP blends.

Sample	Virgin PP	WPCBP	MW0.5	MW1	MW2
V_HB_ (mm/min)	26	42	35	26	18

**Table 5:** Thermal weight loss data of hybrid modified PP blends.

Sample	T_onset 10 wt.%_ (°C)	T_max_ (°C)	Char Residue (wt.%)
PP/WPCBP	317.8	390.6	10.6
PP/MW0.5	318.2	399.2	11.5
PP/MW1	321.4	407.7	10.8
PP/MW2	328.1	418.7	11.4

## Data Availability

Data are contained within the article.
